# Individual Genomic Loci, Transcript Level and Serum Profile of Immune, Antioxidant and Hormonal Markers Associated with Sheep Arthritis

**DOI:** 10.3390/vetsci12020122

**Published:** 2025-02-03

**Authors:** Asmaa Darwish, Ahmed Ateya, Mansour A. Alghamdi, Ahmed El-Sayed

**Affiliations:** 1 Department of Animal Health and Poultry, Animal and Poultry Production Division, Desert Research Center (DRC), Cairo 11753, Egypt; asmaa_vet25@drc.gov.eg; 2 Department of Development of Animal Wealth, Faculty of Veterinary Medicine, Mansoura University, Mansoura 35516, Egypt; 3 Department of Anatomy, College of Medicine, King Khalid University, Abha 62529, Saudi Arabia; m.alghamdi@kku.edu.sa; 4 Genomics and Personalized Medicine Unit, The Center for Medical and Health Research, King Khalid University, Abha 62529, Saudi Arabia

**Keywords:** sheep arthritis, immune response, gene expression, antioxidants, hormones, iron

## Abstract

Arthritis is a leading cause of economic loss in livestock farming including sheep. This study examined the changes in gene expression, antioxidants, pro-inflammatory cytokines, acute-phase proteins (APPs), hormonal assays and iron profiles linked to sheep arthritis, as well as the diagnostic utility of these markers. Blood samples were obtained from 30 apparently healthy rams and 30 rams with arthritis for gene expression and biochemical analyses. Gene expression intensities were much higher in the arthritis-affected rams than in the healthy ones for the genes *IL-1α*, *IL-1β*, *IL-6*, *IL-10*, *TNFα*, *NCF4*, *NFKB*, *TMED*, *FCAMR*, *iNOS* and *COX18*. The *SOD3*, *CAT*, *GPX* and *ATOX1* genes were expressed at substantially lower levels in arthritis-affected rams. Disparities in the nucleotide sequence variants for the amplified DNA bases linked to arthritis for the studied genes were found in the PCR-DNA sequence verdicts of the affected and healthy rams. Immunological, acute-phase protein (APP), antioxidant, hormonal and iron profiles were estimated in both groups and statistically analyzed. The arthritic group in relation to the healthy one showed a significant (*p* < 0.05) increase in pro-inflammatory cytokines, APPs, free radicals, immunoglobulins, cortisol, GH, TSH, ferritin, TIBC and UIBC and a significant (*p* ˂ 0.05) decrease in anti-inflammatory cytokines, antioxidants, complements, insulin, T3, T4, SI, and Tf and Tf sat.% serum levels. The estimated pro-inflammatory cytokines and APPs achieved high values of sensitivity and specificity, positive predictive values (PPVs), negative predictive values (NPVs), a high accuracy rate and a moderate likelihood ratio (LR). The study concluded that ovine arthritis stimulates innate and humeral immunity, resulting in prominent alterations in gene expression, pro-inflammatory cytokines, APP assays and antioxidant profiles, which could be valuable indicators of sheep arthritis.

## Simple Summary

Arthritis is an inflammatory condition that impacts joints’ constituent parts. Immunological and antioxidant indicators have a major role in the generation and regulation of the inflammatory response. In this study, 30 rams with arthritis and 30 rams in good condition were employed for biochemical and gene expression research. The results of this study revealed that there were gene expression, nucleotide sequence and biochemical variations between the healthy and arthritis rams. The shift in the profile of the examined markers points to a potential source of sheep arthritis indications.

## 1. Introduction

According to [1], arthritis is an inflammatory disorder that affects the components of the joints. It can cause a variety of symptoms, including loss of appetite, fever, difficulty standing, increased synovial fluid, joint capsule strains, the weakening of muscles, pain, swelling and lameness. Weight loss, poor reproductive outcomes, lower-quality carcasses, medical expenses and early herd separation owing to disease are all financial losses that result from it [2]. Bacterial arthritis predominates in ruminants, including sheep [3]. The most frequent bacterial species found in sheeps’ afflicted joints are *Erysipelothrix rhusiopathiae*, *Histophilus ovis*, *Escherichia coli* and a few species of *Streptococci* and *Staphylococci* [4]. Although they are less common, viral diseases such as FMD and blue tongue may also be a factor in sheep arthritis [4]. In small ruminants, bacterial infections are more common in the distal joints and can result from contamination, hematogenous seeding, neighboring illness or direct trauma [5]. Sheep arthritis is predisposed by joint injury, ear marking, castration, mulesing, shearing, grazing and dipping [6]. Inadequate management of arthritis in ruminants can lead to significant discomfort that lasts for a long time, reduced joint function, a limited range of motion and even the eventual degeneration of joint, bone and cartilage.

To properly manage the illness and return the joint to a normal physiology, early diagnosis and timely, efficient therapy are crucial. According to [4], it is common practice to use diagnostic imaging, clinical signs and tests of blood and synovial fluid to confirm a diagnosis. A key step in enhancing the precision and effectiveness of care and treatment regimens for sheep with arthritis is learning more about how the immune system responds to the condition and how it changes immune and antioxidant profiles, which will eventually aid in the recovery of the physiology of the afflicted joints [4].

The production and control of the immune inflammatory response are largely mediated by cytokines [7]. Cytokines have a crucial role in regulating the kind, strength and persistence of immune responses by influencing cell development, maturation, activation, proliferation and differentiation [8].

The majority of the acute-phase proteins (APP) in an infected animal’s blood come from the liver, help to maintain homeostasis, prevent pathological damage and stop the growth of microorganisms without the use of antibodies [9]. APP concentrations respond to infection, inflammation and internal or external challenges by either increasing (positive) or decreasing (negative) in plasma. This information is useful for prognostication and diagnosis during infection and inflammation, with notable species-specific variations [10].

When antioxidant resources are depleted and oxidants such as free radicals and reactive oxygen species are abundant, oxidative stress can occur [11]. However, in order to control the excessive creation of free radicals, the animal body possesses antioxidant defense systems [12]. As such, antioxidant enzymes (i.e., catalases) play an important role in these defense mechanisms [13].

Advanced molecular genetic approaches may improve animal health and serve as an auxiliary to disease control [14]. Livestock disease susceptibility and resistance can be accurately predicted by a large number of genetic markers, most of which are single-nucleotide polymorphisms (SNPs) [15]. This suggests that there is variation in the degree of disease susceptibility or resistance among host genotypes [16]. Research on the immune system, antioxidant changes, SNPs, gene expression and sheep arthritis is still in its early stages. Therefore, the purpose of this study was to examine the relationship between sheep arthritis and SNPs, gene expression, the serum profile of immunological and antioxidant marker alterations and other risk factors.

## 2. Materials and Methods

### 2.1. Animals and Study Design

In this study, 60 adult Barki rams from various cities in the Matrouh province of Egypt were used. They ranged in body weight from 50 to 60 kg (mean ± SD: 53.6 ± 3.9) and had an average age of three to four years (mean ± SD: 3.5 ± 0.3). The examined rams were 30 clinically healthy rams and 30 rams that suffered from arthritis. The criteria for inclusion in control group (CG) were having a normal body temperature, pulse, respiration rate, normal appetite, normal movement and intact joints, with no nasal or ocular discharge. Meanwhile, the criteria for the arthritic group (AC) were presence of clinical signs such as lameness in one or more legs (some were unable to stand), swollen joints, carrying the affected leg, high body temperature (40.8 °C), discomfort, pain and poor appetite. A thorough clinical evaluation was conducted on the rams under study, which included taking vital signs such as their heart rate, temperature and respiration rate [17]. Every day, the rams would eat 750 g of concentrate feed mixture (CFM) and 750 g of alfalfa hay in their semi-open, shaded quarters, with water available at all times. The CFM consisted of 300 kg of wheat bran, 250 kg of soy beans, 400 kg of corn, 10 kg of sodium chloride, 20 kg of calcium carbonate, 1 kg of premix, 0.5 kg of Netro-Nill and 0.5 kg of Fylax. In addition to ad libitim water, the rams were given two meals each day: breakfast and dinner. In accordance with the Egyptian Authority Program, the animals were given frequent vaccinations and dewormed.

### 2.2. Blood Sampling and Measurements

The animals in both groups had ten milliliters of blood drawn from their jugular veins, which was then divided into three equal portions. Part one was mixed with EDTA and 5000 I.U. heparin calcium to prevent the blood from clotting. Part two was centrifuged at 3000 r.p.m. for 20 min at 37 °C to obtain serum, and part three was used for DNA and RNA extraction with the EDTA blood. For further biochemical evaluation in the control group (CG) and arthritic group (AG), plasma and serum samples were preserved at −80 degrees Celsius in sterile Eppendorf tubes.

### 2.3. Total RNA Extraction, Reverse Transcription and Quantitative Real-Time PCR

Trizol reagent (RNeasy Mini Ki, Catalogue no. 74104, Tustin, CA, USA) was used to isolate total RNA from ram blood in compliance with the manufacturer’s recommendations. The amount of extracted RNA was quantified and qualified using the NanoDrop^®^ ND-1000 spectrophotometer (UV-Vis spectrophotometer Q5000, Quawell Tech nology, San Jose, CA, USA). Following the manufacturing method (Thermo Fisher, Catalog no. EP0441, London, UK), cDNA was produced for each sample. The gene expression pattern for coding fragments of genes encoding defense (*IL-1α*, *IL-1β*, *IL-6*, *IL-10*, *TNFα*, *NCF4*, *NFKB*, *TMED*, *FCAMR* and *iNOS*) and antioxidants (*SOD3*, *CAT*, *GPX*, *ATOX1* and *COX18*) was assessed using quantitative RT-PCR with SYBR Green PCR Master Mix (2× SensiFastTM SYBR, Bioline, CAT No: Bio-98002, London, UK). The relative mRNA level was determined using real-time PCR using the SYBR Green PCR Master Mix (compatible with Quantitat SYBR Green PCR kit, Catalogue no. 204141).

Primers were designed using the *Ovis aries* sequence that was published in PubMed, as shown in Table 1. The housekeeping gene *ß. actin* was used as a constitutive control for normalization. The reaction mixture, which included total RNA, was prepared using a volume of 25 µL. This PCR master mix contains 12.5 µL of 2× Quantitect SYBR green, 0.5 µL of each primer, 0.25 µL of reverse transcriptase, 3 µL and 4 µL of 5× Trans Amp buffer and 8.25 µL of RNase-free water.

According to Table 1, reverse transcription was carried out for 30 min at 50 °C, followed by 10 min of primary denaturation at 94 °C, 40 cycles of 15 s at 94 °C, 1 min of annealing temperatures and, finally, 30 s at 72 °C. The last step was to place the reaction mixture in a thermal cycler. At the end of the amplification step, a melting curve analysis was performed to confirm the specificity of the PCR product. Using the 2^−ΔΔCt^ technique, we determined the relative expression of each gene in comparison to the ß. actin gene for each sample [18].

### 2.4. DNA Sequencing and Polymorphism Detection

The DNA sequencing process began with the removal of primer dimers, nonspecific bands and any other potential contaminants. According to [19], the purification of real-time PCR products with the expected size (target bands) (Jena Bioscience # pp-201×s/Munich, Ham burg, Germany) was achieved using a PCR purification kit, following the manufacturer’s instructions. In order to ensure that the PCR products were both sufficiently numerous and of high purity and to obtain high yields, a Nanodrop Uv-Vis spectrophotometer (Q5000/Waltham, MA, USA) was employed for protein measurement [20]. According to the enzymatic chain terminator technique created by [21], an ABI 3730XL DNA sequencer (Applied Biosystem, Waltham, MA, USA) was used to discover single-nucleotide polymorphisms (SNPs) in the genes studied in both control and arthritis-affected rams. PCR products bearing the target band were sent for forward and reverse DNA sequencing.

To analyze the DNA sequencing results, the programs Chromas 1.45 and Blast 2.0 were utilized [22]. Variations between the PCR findings of the genes under examination and the reference sequences found in GenBank were categorized as single-nucleotide polymorphisms (SNPs). The amino acid sequence variation of the investigated genes between the rams was determined using the MEGA6 software tool, which is based on data alignment of DNA sequencing [23].

### 2.5. Biochemical Analysis

Through the use of MyBiosecure ELISA kits, the serum concentrations of pro-inflammatory cytokines (IL-1α, IL-1β, IL-6 and TNF-α) and anti-inflammatory cytokines (IL-10) are recorded. Fibrinogen (Fb), serum amyloid A (SAA) and haptoglobin (Hp) concentrations were determined (IBL International Crop ELISA kits (canda)^®^ (Männedorf, Switzerland)). Caeruloplasmin (Cp) concentrations were determined in serum using a turbidimetric technique with kits from Elabscience USA^®^ (Houston, TX, USA). Serum concentrations of free radicals (nitric oxide and malondialdehyde) and antioxidants (catalase, glutathione peroxidase and glutathione reductase) were measured spectrophotometrically using commercial kits from Biodiagnostic^®^ (Worcester, UK). Serum immunoglobulin concentrations (IgG, IgM and IgA) were measured turbidimetrically with kits provided by Biorex Diagnostics^®^ (Antrim, UK). Spectrophotometric analysis of serum iron (SI) and total iron binding capacity (TIBC) was performed using commercial kits from Biodiagnostic Company^®^ (Worcester, UK). Levels of complement-3 (C3) and complement-4 (C4) in serum were measured by the enzyme-linked immunosorbent assay (ELISA) method using New Test Company^®^ commercial kits (Hangzhou, China). Chemiluminescence immunoassay (CLIA) kits supplied by Diasorin^®^ (Saluggia, Italy) were used for serum hormonal tests, which included measurements of growth hormone (GH), insulin, cortisol, TSH, T3 and T4. Transcriptional factor (Tf) concentrations in serum were determined with turbidimetric technique using Elabscience USA^®^ assays. Iron concentrations in serum were determined using the chemiluminescence immunoassay (CLIA) technique with Abnova^®^ (Taipei, Taiwan) diagnostic kits.

Transferrin saturation percentage (Tf sat %) = SI (Serum iron)/TIBC (Total iron capacity) × 100

Unsaturated iron binding capacity (UIBC) = SI − TIBC

### 2.6. Statistical Analysis

The rams that were being studied had their SNP distribution for the indicated genes analyzed using Fisher’s exact test, which yielded a significant result (*p* < 0.01). The mean ± standard deviation (SD) was used to represent the statistical parameters. We used SPSS program version 23 to statistically analyze the data. We used an independent-sample T-test to compare the means of the groups that were tested. We utilized Pearson’s simple correlation method to find the relationships between the genetic, immunological, APP and antioxidant characteristics. At a significance level of *p* < 0.05, a difference was deemed to exist.

− GraphPad Prism version 5 was used to estimate the cut-off points, sensitivity, specificity and likelihood ratio (LR) for the measured cytokines and APPs between the two groups.
− The following equations were used to compute the positive predictive value (PPV), negative predictive value (NPV), accuracy rate (AR) and percentages of increase for these variables:

PPV=True positive÷Total positive×100.

NPV=True negative÷Total negative×100.

$$Accuracy\;rate=\left(True\;positive+True\;negative\right)÷Total\;population\times 100.$$

$$\begin{array}{c} Percentage\;of\;increaese\\ \;\;\;\;\;\;\;\;\;\;\;\;\;\;\;\;\;\;=(The\;mean\;value\;of\;the\;marker\;concentration\;in\;AG\\ \;\;\;\;\;\;\;\;\;\;\;\;\;\;\;\;\;\;-The\;mean\;value\;of\;its\;concentration\;in\;CG)\\ \;\;\;\;\;\;\;\;\;\;\;\;\;\;\;\;\;\;÷The\;mean\;value\;of\;its\;concentration\;in\;CG\times 100. \end{array}$$

## 3. Results

### 3.1. Clinical Examination

The clinically healthy rams showed a normal body temperature, pulse, respiration rate, lack of nasal or ocular discharge, normal appetite, normal mobility and intact joints. Alternatively, the rams with arthritis showed signs of lameness in one or more legs (some were not even able to stand up), had swollen joints, carried the affected leg, and had an elevated body temperature (40.8 °C), pain, discomfort and a lack of appetite.

### 3.2. Patterns for Transcript Levels of Immune and Antioxidant Indicators

**Figure 1:**
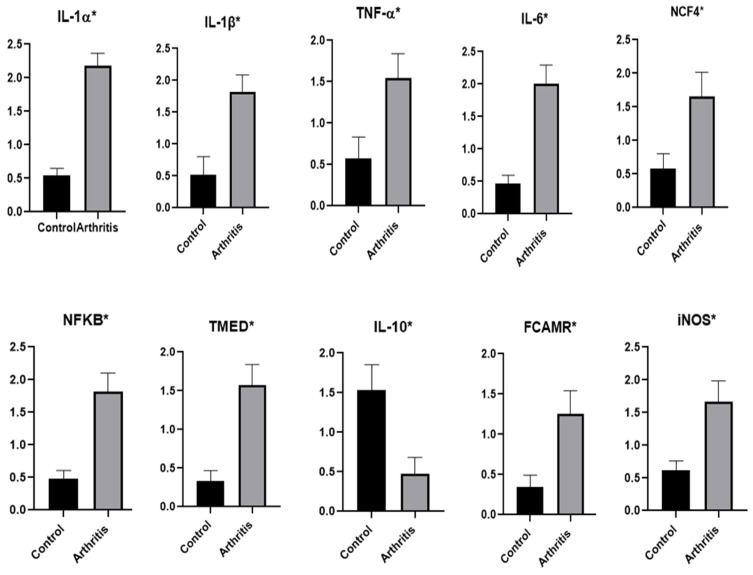
Rams with arthritis and normal rams had different immune gene transcript levels. A significance level of *p* < 0.05 is indicated by the symbol *.

**Figure 2:**
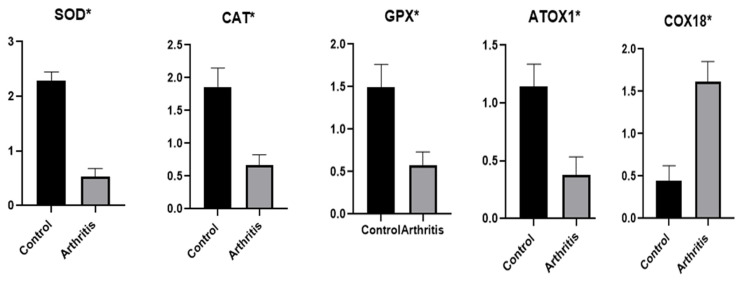
Rams with arthritis and normal rams had different antioxidant gene transcript levels. A significance level of *p* < 0.05 is indicated by the symbol *.

Figure 1 and Figure 2 showed the transcript profiles for the evaluated antioxidant and immunological indicators. The rams with arthritis showed significantly greater levels of gene expression for the genes *IL-1α*, *IL-1β*, *IL-6*, *IL-10*, *TNFα*, *NCF4*, *NFKB*, *TMED*, *FCAMR*, *iNOS* and *COX18* than did the healthy rams. The expression of the *SOD3*, *CAT*, *GPX* and *ATOX1* genes was significantly reduced in the rams with arthritis.

The highest possible level of mRNA for the arthritic rams was 2.18 ± 0.18 for *IL-1α*, while the lowest amount of each gene was 0.37 ± 0.09 for *ATOX1*. TMED had the lowest possible level of mRNA (0.33 ± 0.08), while *SOD3* had the highest (2.29 ± 0.08) among all the genes examined in the healthy rams.

### 3.3. Genetic Polymorphisms of Immune and Antioxidant Genes

*IL-1α* (466 bp), *IL-1β* (361 bp), *IL-6* (370 bp), *IL-10* (387 bp), *TNFα* (386 bp), *NCF4* (345 bp), *NFKB* (411 bp), *TMED* (408 bp), *FCAMR* (378 bp), *iNOS* (480 bp), *SOD* (381 bp), *CAT* (473 bp), *GPX* (416 bp), *ATOX1* (430 bp) and *COX18* (415 bp) PCR-DNA sequence judgments of healthy and affected rams showed variations in the amplified DNA bases linked to arthritis. Using the reference gene sequences from GenBank and the DNA sequence variations between the immunological and antioxidant indicators examined in the studied rams, all of the identified SNPs were accepted (Figures S1–S15).

The single base difference distribution for immunological and antioxidant indicators differed significantly (*p* < 0.01) between the healthy and arthritis-affected rams, according to Fisher’s exact analysis. Table 2 showed that all of the immunological and antioxidant markers that were investigated had exonic region changes when comparing the coding DNA sequences of the affected and healthy rams. Eleven of the thirty-one SNPs found in the immunological and antioxidant genes were synonymous, whereas the other twenty-one were non-synonymous.

For the immune indicators of the *IL-1α* gene (466 bp), DNA sequencing was applied to discover three recurring SNPs; two of them, C185T and T191C, led to non-synonymous mutations: P62L and M64T, respectively. By contrast, the 121T amino acid was noted to be a result of the synonymous mutation A363C. The *IL-1β* gene (361 bp) enclosed four SNPs. Three SNPs were non-synonymous: C29T, C46A and T89C, which produced the amino acids S10L, A16T and L30P for exchange. Meanwhile, the synonymous SNP A58C was linked to amino acid 20R. Upon analyzing the 370 bp sequence of the *IL-6* gene, a recurrent SNP was discovered, C341T SNP, which resulted in the non-synonymous mutation T114I. When the 386 bp DNA sequence of the *TNFα* gene was analyzed, one recurrent SNP was discovered: C242T, which carried the non-synonymous mutation T81M. Two repetitive SNPs were found when the *IL-10* (387 bp) gene was sequenced. T39C involved the synonymous mutation 13R, and G219C involved the non-synonymous mutation that changed the amino acid R37S.

Two frequently occurring synonymous SNPs, C66T and A264G, were found in the 411 bp DNA structure of the *NFKB* marker and were linked to amino acids 22A and 88K, respectively. Five regular SNPs in the *NCF4* gene (345 bp) were found by DNA sequencing; four of these, G36T, C243T, G261A and A267G, resulted in synonymous changes of 12P, 81F, 87V and 89T, respectively. However, the non-synonymous mutation brought about by C38A SNP resulted in the production of amino acid R13K. The *TMED1* gene (408 bp) contains three repeating SNPs that were identified by DNA sequencing; two of them, G125C and T337C, produced non-synonymous mutations, G42A and W133L, respectively. On the other hand, amino acid 49V was produced by the synonymous mutation caused by SNP G147A. One recurrent SNP was identified during the 378 bp sequencing of the FCAMR gene, in which the synonymous mutation 90L was induced by the C268T SNP. When the 480 bp DNA sequence of the *iNOS* gene was analyzed, three recurrent SNPs were discovered: C38T, C77T and T410C, which carried the non-synonymous mutations S13L, S26L and M137T.

In terms of antioxidant markers, recurring non-synonymous SNPs were identified in the nucleotide categorization of *SOD* (381 bp), *CAT* (473 bp), *ATOX1* (430 bp) and *COX18* (415 bp) genes: S49R, T52M, R128P and V37A, respectively. These altered amino acids were caused by the C147G, C155T, G383C and T110C SNPs, respectively. Two repetitive SNPs were found when the *GPX* (416 bp) gene was sequenced. G193A and C298T involved the non-synonymous mutations H65N and R100C.

### 3.4. Biochemical Profile

This study proved that sheep arthritis triggered a robust immune response, as shown by the significantly higher levels of pro-inflammatory cytokines (IL-1α, IL-1β, IL-6 and TNF-α), acute-phase proteins (Fb, Hp, SAA and Cp) and free radicals (NO and MDA) and the significantly lower levels of anti-inflammatory cytokines (IL-10), antioxidants (CAT, GR and GPx) and complements (C3 and C4) in AG compared to CG. Furthermore, blood cortisol, GH, TSH, ferritin, TIBC and UIBC levels were significantly higher (*p* < 0.05) in the AG compared to the CG, but serum insulin, T3, T4, SI, Tf and Tf sat. % levels were significantly lower (*p* ˂ 0.05) (Table 3).

The estimated cytokines and APPs in the arthritic sheep had a high sensitivity, specificity, PPV and accuracy rate at AUC = 1, as well as moderate LRs (5–10). Among them, Hp had the highest percentage of increase, suggesting that it is important for both diagnosis and prognosis (Table 4).

### 3.5. Correlation Between Gene Expression Patterns and Serum Profiles of Immunological, APP and Antioxidant Markers

The mRNA levels of *IL1-α* were positively correlated with the serum level of MDA, TNFα and NO (r = 0.997, *p* = 0.04; r = 1, *p* = 0.001; and r = 0.998, *p* = 0.03), respectively. There was a positive correlation between the mRNA levels of IL1-β and the serum levels of CAT, IL6, IL1-β, Hp, Cp, Fb and GPx (r = 1, *p* = 0.002) and between MDA and NO (r = 1, *p* = 0.009, r = 0.999, *p* = 0.02), respectively. A positive correlation (r = 0.998, *p* = 0.04) was seen between the iNOS mRNA levels and the blood GSH level.

## 4. Discussion

The purpose of this study was to confirm that single-nucleotide polymorphisms (SNPs), changes in immunological and antioxidant markers, APP gene expression and serum profiles, and other similar variables can be used to diagnose arthritis in Barki rams. The prevalence of lameness in ruminants is a significant factor in the declining profitability of livestock husbandry. It is the second most common cause of animal slaughter after mastitis and low fertility [24]. Other indirect costs of lameness include decreased milk production, milk fat, protein and treatment expenses [25]. The arthritic rams had lameness in one or more legs, fever, emaciation, swollen joints, discomfort, pain and decreased appetite, according to a clinical examination. The aforementioned clinical indicators have been additionally detailed by [1,26] in sheep and [6] in calves.

We were able to compare the immune and antioxidant states of healthy rams to those of arthritic rams by analyzing the mRNA levels of antioxidant (*SOD3*, *CAT*, *GPX*, *ATOX1* and *COX18*) and immunological (*IL-1α*, *IL-1β*, *IL-6*, *IL-10*, *TNFα*, *NCF4*, *NFKB*, *TMED*, *FCAMR* and *iNOS*) genes. When comparing healthy rams to those with arthritis, the expression levels of the following genes were noticeably higher: *IL-1α*, *IL-1β*, *IL-6*, *IL-10*, *TNFα*, *NCF4*, *NFKB*, *TMED*, *FCAMR*, *iNOS* and *COX18*. The rams suffering from arthritis exhibited abnormally low levels of gene expression for *SOD3*, *CAT*, *GPX* and *ATOX1*. No study has ever looked at the amounts of antioxidant and immunological markers in sheep transcripts in relation to arthritis. Consequently, quantitative changes in the expression of the relevant genes occur prior to the development of arthritis.

This research described the antioxidant (*SOD3*, *CAT*, *GPX*, *ATOX1* and *COX18*) and immunological (*IL-1α*, *IL-1β*, *IL-6*, *IL-10*, *TNFα*, *NCF4*, *NFKB*, *TMED*, *FCAMR* and *iNOS*) genes in rams that had arthritis and those that did not. The PCR-DNA sequencing method was utilized for this purpose. The findings point to variations in the SNPs affecting the two groups. It is worth noting that the polymorphisms found and made available in this context provide more information for the evaluated metrics compared to similar datasets collected from GenBank. No studies have investigated whether or not single-nucleotide polymorphisms (SNPs) in antioxidant and immunological genes increase the incidence of arthritis in sheep. The gene sequences of *Ovis aries*, which were published in PubMed as part of our study, are the first to demonstrate this association.

Indirect indicators of inflammation include cytokines such as NFKB, IL-1α, TNF-α, IFN-H, IL-1B and IL-6 [27]. Macrophages inhibit the NK cell production of IL-1 and TNF, while the anti-inflammatory cytokine IL-10 stops Th1 lymphocytes from making IFN- and IL-2 [28,29]. The reason why there is a noticeable rise in the number of uterine washings performed on cows with subclinical endometritis could be because IL-10, which is produced by different types of T-cells, has anti-inflammatory properties. These properties protect the uterine tissues from the harmful effects of inflammatory cells and mediators through its interaction with suppressor T CD8+ cells [30]. However, IL-10 has been associated with inflammation, protracted postpartum infection and damage to uterine local resistance [31]. It appears that the innate immunity gene neutrophil cytosolic factor 4 (NCF4) is associated with and has an effect on mastitis in cattle [32].

One member of the family of transmembrane proteins that transport proteins across vesicles is known as transmembrane P24 trafficking protein 1 (TMED1) [33]. In goats, the *TMED1* gene has shown a significant increase in expression in kids with diarrhea compared to those without diarrhea [34]. It is possible that FCAMR is involved in the immunological response to microbes mediated by IgA and IgM [35]. Ewes with endometritis had a marked increase in the expression of the *FCAMR* gene [35]. From the amino acid L-arginine, nitric oxide (NO) is produced by nitric oxide synthase (NOS). NO is crucial for many physiological and pathological processes. The transcription of the *NOS2A* gene, which encodes iNOS, is regulated by inflammatory mediators released by immunocompetent cells such as macrophages and neutrophils [36].

Antioxidants can shield us from harm by removing reactive oxygen species (ROS) from the air, blocking their production or preserving transition metals, which are essential for free radical production [37]. Catalase (CAT) and superoxide dismutase (SOD), which are the body’s own enzymatic and non-enzymatic antioxidant defenses, are known as endogenous antioxidant markers [38]. Mitochondrial cytochrome c oxidase assembly components are encoded by the *COX18* gene and are necessary for the insertion of integral membrane proteins into the inner mitochondrial membrane. It plays an essential role in cytochrome c oxidase assembly and function as well [39]. The *COX18* gene was much less expressed in cows with mastitis as compared to healthy Holstein and Montbéliarde dairy cows [40].

Pathogen invasion may be a potent oxidizing stimulus that sets off immunological responses to counter the pathogen onslaught by boosting the activity of neutrophils and macrophages. This leads to the production and accumulation of an excessive amount of ROS, which ultimately results in oxidative stress [6]. The considerable change in the expression configuration of antioxidant (*SOD3*, *CAT*, *GPX*, *ATOX1* and *COX18*) and immunological (*IL-1α*, *IL-1β*, *IL-6*, *IL-10*, *TNFα*, *NCF4*, *NFKB*, *TMED*, *FCAMR* and *iNOS*) indicators in rams with arthritis may be explained by the previously described factors. Therefore, we believe that the arthritis in the rams in the study is caused by an infectious etiology. Our real-time PCR data revealed that the rams with arthritis also displayed a significant inflammatory response.

Cytokines are significant inflammatory response modulators. It is possible to classify these glycoprotein molecules as either pro- or anti-inflammatory. When infections occur, pro-inflammatory cytokines are released quickly, alerting immune cells to start and spread the immune response [14,15,41]. Conversely, anti-inflammatory cytokines regulate and resolve inflammatory actions, preventing excessive damage. In this study, the observed upregulation of pro-inflammatory cytokines and downregulation of anti-inflammatory cytokines in the AG corresponded to an exacerbated inflammatory response. These findings align with previous reports [14,15,16,41], which documented elevated systemic and local pro-inflammatory cytokines and diminished anti-inflammatory cytokines in arthritis, triggering joint damage, impaired mobility and functional decline. Targeting pro-inflammatory cytokines has been recommended as a therapeutic approach to mitigate chronic-arthritis-induced damage [42].

APPs are non-specific innate immune molecules that are rapidly secreted into circulation during infection and surgery and persist in the blood for a long time [43]. Their pronounced increment in the AG in our research was necessary to maintain body homeostasis and limit microbial growth until the synthesis of immunoglobulins. In addition, they have potent anti-inflammatory, anti-bacterial and antioxidant actions [44]. These findings were concurrent with previous studies that suggested APPs as an auxiliary tool in the diagnosis of different diseases in ruminants [44,45]. According to [46], the pronounced acute-phase reactant levels were useful for disease activity evaluations and predictions of outcomes.

Another important factor is free radicals, which are extremely reactive chemicals created during immunological reactions. In order to neutralize pathogens, they oxidize vital components such as DNA and proteins [47]. In the current work, the AG showed significant oxidative stress, which is defined by an excess of free radicals, a depletion of antioxidants and subsequent damage to chondrocytes and other tissues. Similar oxidative imbalances have been reported in both animal and human inflammatory diseases, including arthritis [6,24,48].

As a key component of immune defense, the complement system facilitates tissue repair, immune complex clearance and antigen recognition [25,49]. This study’s notable decrease in C3 and C4 levels in the AG suggested chronic inflammation and hyperactivation of the complement system, which is responsible for consuming complement system proteins [27]. There is no doubt that this complement element deficiency worsens the conditions of the rams in the AG group and predisposes them to other infections and inflammatory disorders [28]. However, this study’s increased levels of IgA, IgM and IgG in the AG suggest a strong humoral response powered by pro-inflammatory cytokines [29]. These results support the use of immunoglobulin titers to assess the course and activity of disease [30]. Similarly, [29] and [30] recorded raised serum IgA, IgM and IgG in arthritic patients.

The current study found that different endocrine responses are triggered by arthritis. Serum levels of cortisol, growth hormone (GH) and thyroid-stimulating hormone (TSH) were higher in animals with arthritis (AG), whereas serum levels of insulin, triiodothyronine (T3) and thyroxine (T4) were lower. These hormonal changes are comparable to those seen in other inflammatory conditions [31,32]. The primary cause of the observed alterations is hypoglycemia, which is a prevalent characteristic of inflammatory conditions like arthritis. Significant energy is required for pro-inflammatory activation and immune responses, which causes marked hypoglycemia in afflicted animals. In order to combat hypoglycemia, energy-regulating glands, especially the pituitary gland, become active and secrete more growth hormone (GH) and adrenocorticotropic hormone (ACTH) [33,34]. The adrenal glands are stimulated by ACTH to release cortisol into the bloodstream [33,34]. Cortisol and GH both lower the cell uptake of glucose, which leads to hyperglycemia. Furthermore, cortisol causes hyperglycemia by suppressing thyroid gland T3 and T4 secretion, increasing hepatic glycogenolysis and gluconeogenesis and encouraging insulin resistance [31,32]. Concurrently, pancreatic beta cells decrease insulin synthesis in response to hypoglycemia, which further reduces the cell uptake of glucose [31,32]. Consequently, the arthritis group (AG) in this study showed significant hypercortisolemia, hyperinsulinemia, decreased T3 and T4 levels and increased GH levels. The increased TSH levels in AG are likely a compensatory response by the pituitary gland to the reduced serum concentrations of T3 and T4 [31,32]. These results demonstrate a synchronized hormonal response intended to maintain immunological function in inflammatory conditions, improve gluconeogenesis and counteract hypoglycemia [31,32,33,34].

This study’s iron profile for the AG showed significant changes in iron metabolism such as hypoferremia, hyperferritinemia and hypotransferrinemia. The authors of [35,36] discovered similar alterations in rheumatoid arthritis (RA) patients’ iron profiles. They reasoned that the inflammatory immune response that accompanied the illness was responsible for these changes. However, activated pro-inflammatory cytokines reduce iron bioavailability by promoting ferritin storage, releasing hepcidin, which prevents the intestinal absorption of iron, and reducing transferrin activity, which prevents infections from spreading [14,15,41]. In chronic inflammatory circumstances, its activity can produce anemic swings in unhealthy people, despite its antimicrobial properties [14,15,41]. Similarly to how ferritin is a positive acute-phase protein and transferrin is a negative one, these alterations were also influenced by the acute-phase response that was shown in the AG [44,45]. In addition, the host erythrocytes are attacked by oxidative stress and unregulated free radicals throughout the disease course, leading to their lysis and excess iron release. The oxidative action of free iron causes the body to boost ferritin synthesis as a defense mechanism [24,48]. In a logical sense, the exceptional hypoferremia caused TIBC and UIBC to rise while the Tf sat.% in the AG fell.

According to Table 4, APPs and estimated pro-inflammatory cytokines in ovine arthritis can be used as biomarkers for sheep arthritis, particularly IL-1α, IL-6, TNF-α and Hp, which had the best specificity, LR, PPV and accuracy rates. But out of all of them, Hp was given precedence due to the degree of its increase. Several writers have already found similar results in various animal and human diseases. They validated the significance of pro-inflammatory cytokines and APPs in the diagnosis and monitoring of inflammatory disorders [14,15,16,41].

Our study was limited by the lack of arthrocentesis in the afflicted joints to evaluate the cause of joint inflammation, identify the content of inflammatory infiltrates and measure the viscosity and turbidity of the synovial fluid. Therefore, future research must consider arthrocentesis in the affected joints.

## 5. Conclusions

Our findings highlight the significance of the nucleotide sequence variation and expression profiles of the inspected immune and antioxidant genes as predisposing factors and monitors their relationship to arthritis predisposition in sheep. It can also be concluded that ovine arthritis evokes innate as well as humeral immunity. The pro-inflammatory cytokines, APPs, antioxidants, hormonal assays and iron profile changes are a reflection of the immune response in sheep with arthritis.

## Figures and Tables

**Table 1:** Forward and reverse oligonucleotide-based real-time PCR primers for the immunological and antioxidant genes being investigated.

Investigated Marker	Primer	Product Size (bp)	Annealing Temperature (°C)	GenBank Isolate	Origin
*IL-1α*	F5′-AGAAGTCCTTCTATGATGCAAG-3′ R5′-GAGATTCTTAGAGTCACAGGA-3′	466	60	NM_001009808.1	Present Research
*IL-1β*	F5′-CAGCCATGGCAACCGTACCTG-3′ R5′-ACTGACTGCACGGCTGCATCAC-3′	361	60	NM_001009465.2	
*IL-6*	F5′-GTAGTTCCTGGGCATTCCCTC-3′ R5′-CAGCCTAAACATATAAATACA-3′	370	58	NM_001009392.1	
*TNFα*	F5′-CCTGCCGGAATACCTGGACTAT-3′ R5′-CTCAAGGAACGTTGCGAAGTAT-3′	386	60	NM_001024860.1	
*IL10*	F5′-CTGAAGACCCTCCGGCTGCGGC-3′ R5′-TCACAGAGAAGCTCAGTAAATA-3′	387	58	NM_001009327.1	
*NFKB*	F5′-ATCCACCTGCACGCACACAGC-3′ R5′-GCTGTCATAGATGGCGTCCGAC-3′	411	60	XM_060416845.1	
*NCF4*	F5′-AGAGGCAGCTCCTGGGGACTC-3′ R5′-GAAGCGCTCCTCCAGCTTGCT-3′	345	60	XM_042247241.2	
*TMED1*	F5′-AGCACTGGCTGGCTTGCAGGT-3′ R5′-GTGACTGTTCTGGCAAGAACAC-3′	408	58	XM_060415483.1	
*FCAMR*	F5′-GAGGCTTGTTCGTGGTGAGGCT-3′ R5′-CGGAGCCCTCTGCCTGGCTGC-3′	378	58	XM_042257020.1	
*iNOS*	F5′-CAGGAACCTACCAGCTGACGG-3′ R5′-GGTCGCGGCCGTCAGCCTGCA-3′	480	60	AF223942.1	
*SOD*	F5′-ATCCGCGACATGCACGCCAAG-3′ R5′-CCAGACCTGGCCATCTCGCAC-3′	381	60	XM_027970902.2	
*CAT*	F5′-CTGATGTCCTGACCACTGGCGC-3′ R5′-CATGTCCGGATCCTTCAGGTG-3′	473	58	XM_060400055.1	
*GPX*	F5′-AGCTCACTGCTCTCAACTTGG-3′ R5′-AGAGCGGATGCGCCTTCTCGC-3′	416	58	XM_004018462.5	
*ATOX1*	F5′-CGTTGCTGTCGGAGGCGTAGTC-3′ R5′-CGGCTTGACTTTATTGCAGGGA-3′	430	58	NM_001009429.1	
*COX18*	F5′-ATGCGCGTAGAGGCCTCGGCG 3′ R5′-GATGTAGTGCTGGTAGGCAGC-3′	415	60	XM_027971062.3	
*ß. actin*	F5′-AATTCCATCATGAAGTGTGAC-3′ R5′-GATCTTGATCTTCATCGTGCT-3′	150	58	KU365062.1	

**Table 2:** Immunological and antioxidant marker distribution in healthy and arthritic rams with a single base difference and possible genetic change.

Gene	SNPs	Healthy *n* = 30	Arthritic *n* = 30	Total *n* = 60	Kind of Inherited Change	Amino Acid Order and Sorting
*IL-1α*	C185T	-/30	18/30	31/60	Non-synonymous	62 P to L
	T191C	21/30	-/30	21/60	Non-synonymous	64 M to T
	A363C	13/30	-/30	13/60	Synonymous	121 T
*IL-1β*	C29T	19/30	-/30	19/60	Non-synonymous	10 S to L
	C46A	-/30	23/30	23/60	Non-synonymous	16 A to T
	A58C	11/30	-/30	11/60	Synonymous	20 R
	T89C	14/30	-/30	14/60	Non-synonymous	30 L to P
*IL-6*	C341T	17/30	-/30	17/60	Non-synonymous	114 T to I
*TNFα*	C242T	-/30	24/30	24/60	Non-synonymous	81 T to M
*IL10*	T39C	-/30	14/30	14/60	Synonymous	13 R
	G219C	-/30	19/30	19/60	Non-synonymous	73 R to S
*NFKB*	C66T	13/30	-/30	13/60	Synonymous	22 A
	A264G	22/30	-/30	22/60	Synonymous	88 K
*NCF4*	G36T	-/30	15/30	15/60	Synonymous	12 P
	G38A	17/30	-/30	17/60	Non-synonymous	13 R to K
	C243T	-/30	19/30	19/60	Synonymous	81 F
	G261A	-/30	24/30	24/60	Synonymous	87 V
	A267G	15/30	-/30	15/60	Synonymous	89 T
*TMED1*	G125C	12/30	-/30	12/60	Non-synonymous	42 G to A
	G147A	14/30	-/30	14/60	Synonymous	49 V
	T337C	22/30	-/30	22/60	Non-synonymous	113 W to L
*FCAMR*	C268T	-/30	14/30	14/60	Synonymous	90 L
*iNOS*	C38T	19/30	-/30	19/60	Non-synonymous	13 S to L
	C77T	18/30	-/30	18/60	Non-synonymous	26 S to L
	T410C	-/30	15/30	15/60	Non-synonymous	137 M to T
*SOD*	C147G	-/30	22/30	22/60	Non-synonymous	49 S to R
*CAT*	C155T	19/30	-/30	19/60	Non-synonymous	52 T to M
*GPX*	C193A	10/30	-/30	10/60	Non-synonymous	65 H to N
	C298T	23/30	-/30	23/60	Non-synonymous	100 R to C
*ATOX1*	G383C	-/30	17/30	17/60	Non-synonymous	128 R to P
*COX18*	T110C	21/30	-/30	21/60	Non-synonymous	37 V to A

Fisher’s exact analysis revealed that the immune and antioxidant markers’ single base difference distribution varied significantly (*p* < 0.01) between rams with and without arthritis. A = Alanine; C = Cysteine; F = Phenylalanine; G = Glycine; H = Histidine; I = Isoleucine; K = Lysine; L = Leucine; M = Methionine; N = Asparagine; P = Proline; R = Arginine; S = Serine; T = Threonine; V = Valine; and W = Tryptophan.

**Table 3:** Immunological, APP, antioxidant, hormonal assay and iron profiles of arthritic group (AG) compared to control (CG); values are means ± SD.

Parameters	CG	AG
IL-1α (Pg/mL)	24.05 ± 3.34	95.73 ± 1.78 *
IL-1β (Pg/mL)	25.99 ± 2.96	99.14 ± 0.64 *
IL-6 (Pg/mL)	24.63 ± 2.92	86.38 ± 0.08 *
TNF-α (Pg/mL)	24.91 ± 2.98	87.24 ± 1.70 *
IL-10 (Pg/mL)	103.70 ± 3.31	90.69 ± 2.26 *
IgG (mg/dL)	229.02 ± 19.74	350.26 ± 36.38 *
IgM (mg/dL)	13.79 ± 1.43	41.43 ± 5.95 *
IgA (mg/dL)	4.49 ± 0.83	8.62 ± 0.66 *
C3 (mg/dL)	151.17 ± 5.29	110.68 ± 1.74 *
C4 (mg/dL)	12.07 ± 0.60	7.14 ± 0.83 *
Fb (mg/dL)	122.01 ± 8.49	225.01 ± 3.70 *
Cp (mg/dL)	2.30 ± 1.15	6.24 ± 0.03 *
Hp (g/dL)	0.15 ± 0.02	2.59 ± 0.49 *
SAA (mg/L)	2.32 ± 0.15	6.94 ± 0.16 *
MDA (nmol/mL)	12.95 ± 1.14	23.70 ± 1.52 *
NO (μmol/L)	26.80 ± 1.20	32.91 ± 1.78 *
GSH (ng/mL)	8.15 ± 0.62	5.06 ± 0.51 *
CAT (U/L)	412.25 ± 14.64	289.20 ± 6.16 *
GPx (mU/L)	1015.45 ± 2.95	745.01 ± 15.65 *
Cortisol (μg/dL)	1.79 ± 0.16	6.44 ± 0.06 *
Insulin (μIU/mL)	8.41 ± 0.15	7.09 ± 0.17 *
T3(ng/mL)	1.74 ± 0.15	1.02 ± 0.01 *
T4 (µg/mL)	0.85 ± 0.08	0.64 ± 0.02 *
TSH (µIU/mL)	0.010 ± 0.002	0.022 ± 0.012 *
GH (ng/dL)	12.39 ± 1.47	16.80 ± 0.06 *
SI (μg/dL)	106.89 ± 2.46	89.36 ± 1.57 *
TIBC (μg/dL)	327.39 ± 2.16	341.06 ± 3.20 *
UIBC (μg/dL)	220.50 ± 2.24	251.69 ± 2.74 *
Transferrin(mg/dL)	124.65 ± 2.74	86.54 ± 0.24 *
Tf sat.%	32.65 ± 0.66	26.20 ± 0.39 *
Ferritin (ng/mL)	13.60 ± 1.05	19.54 ± 0.53 *

IL1-α Interleukin 1 alpha; IL1-β Interleukin 1 beta; IL6 Interleukin 6; TNF-α Tumor necrosis factor-alpha; IL10 Interleukin 10; Fb Fibrinogen; Cp Caeruloplasmin; Hp Haptoglobin; SAA Serum amyloid A; MDA Malondialdhyde; NO Nitric oxide; GSH Glutathione reductase; CAT Catalase; GPx Glutathione peroxidase. Statistical significance between AG and CG is indicated by (*), when *p* < 0.05. IgG Immunoglobulin G; IgM Immunoglobulin M; IgA Immunoglobulin A; C3 Complement-3; C4 Complement-4; T3 Triiodothyronine; T4 Thyroxine; TSH Thyroid stimulating hormone; GH Growth hormone; SI Serum iron; TIBC Total iron capacity; UIBC Unsaturated iron binding capacity; Tf Transferrin.

**Table 4:** Cut-off points, sensitivity, specificity, LR, PPV, NPV, accuracy rate and percentages of increase or decrease in interleukins and APPs in AG compared to CG.

Parameter	IL-1α (Pg/mL)	IL-1β (Pg/mL)	IL-6 (Pg/mL)	TNF-α (Pg/mL)	IL-10 (Pg/mL)	Fb (mg/dL)	SAA (mg/L)	Hp (g/dL)	Cp (mg/dL)
Cut-off	29.50	28.5	28.5	28.5	100.80	132.5	2.45	0.185	3.60
Sensitivity	100%	100%	100%	100%	100%	100%	100%	100%	100%
Specificity	90%	85%	90%	90%	80%	85%	80%	90%	80%
LR	10	6.67	10	10	5	6.67	5	10	5
PPV	90.91%	86.96%	90.91%	90.91%	83.33%	86.96%	83.33%	90.91%	83.33%
NPV	100%	100%	100%	100%	100%	100%	100%	100%	100%
Accuracy rate	95%	92.5%	95%	95%	90%	92.5%	90%	95%	90%
% increase or decrease	298.05%	281.45%	226.35%	250.22%	−12.57%	84.41%	199.14%	1626.67%	171.30%

IL1-α Interleukin 1 alpha; IL1-β Interleukin 1 beta; IL6 Interleukin 6; TNF-α Tumor necrosis factor-alpha; IL10 Interleukin 10; Fb Fibrino gen; SAA Serum amyloid A; Hp Haptoglobin; Cp Caeruloplasmin; LR: Likelihood ratio, PPV: Positive predictive value, NPV: Negative predictive value. LR = 0.5–5: low; LR = 5–10: moderate; LR > 10: high.

## Data Availability

Upon reasonable request, the appropriate author will share supporting data for the study’s conclusions.

## Supplementary Materials

The following supporting information can be downloaded at: https://www.mdpi.com/article/10.3390/vetsci12020122/s1, Figure S1: Representative Sequence alignment of *IL-1α* gene (466 bp) between healthy (H), and arthritis (A) rams. Figure S2: Representative Sequence alignment of *IL-1β* gene (361 bp) between healthy (H), and arthritis (A) rams. Figure S3: Representative Sequence alignment of *IL-6* gene (370 bp) between healthy (H), and arthritis (A) rams. Figure S4: Representative Sequence alignment of *TNFα* gene (386 bp) between healthy (H), and arthritis (A) rams. Figure S5: Representative Sequence alignment of *IL-10* gene (387 bp) between healthy (H), and arthritis (A) rams. Figure S6: Representative Sequence alignment of *NFKB* gene (411 bp) between healthy (H), and arthritis (A) rams. Figure S7: Representative Sequence alignment of *NCF4* gene (345 bp) between healthy (H), and arthritis (A) rams. Figure S8: Representative Sequence alignment of *TMED1* gene (408 bp) between healthy (H), and arthritis (A) rams. Figure S9: Representative Sequence alignment of *FCAMR* gene (378 bp) between healthy (H), and arthritis (A) rams. Figure S10: Representative Sequence alignment of *iNOS* gene (480 bp) between healthy (H), and arthritis (A) rams. Figure S11: Representative Sequence alignment of *SOD* gene (381 bp) between healthy (H), and arthritis (A) rams. Figure S12: Representative Sequence alignment of *CAT* gene (473 bp) between healthy (H), and arthritis (A) rams. Figure S13: Representative Sequence alignment of *GPX* gene (416 bp) between healthy (H), and arthritis (A) rams. Figure S14: Representative Sequence alignment of *ATOX1* gene (430 bp) between healthy (H), and arthritis (A) rams. Figure S15: Representative Sequence alignment of *COX18* gene (415 bp) between healthy (H), and arthritis (A) rams.
