# Supplementary material for: Individual Genomic Loci, Transcript Level and Serum Profile of Immune, Antioxidant and Hormonal Markers Associated with Sheep Arthritis
Source: Vet Sci. 2025 Feb 3;12(2):122. doi: 10.3390/vetsci12020122 (PMC11861797; doi:10.3390/vetsci12020122)
Supplement: Supplementary file 1 [file vetsci-12-00122-s001.zip › vetsci-3422464-supplementary.pdf]

```
NM_001009808.1      AGAAGTCCTTCTATGATGCAAGCTATGAGCCACTTCGTGAGGACCACATGAATAAGTTTA   60
H                   AGAAGTCCTTCTATGATGCAAGCTATGAGCCACTTCGTGAGGACCACATGAATAAGTTTA   60
A                   AGAAGTCCTTCTATGATGCAAGCTATGAGCCACTTCGTGAGGACCACATGAATAAGTTTA   60
                    ************************************************************

NM_001009808.1      TGTCCCTGGATACCTCGGAAACCTCCAAGACATCCAGGCTTAGCTTCAAGGAGAATGTGG   120
H                   TGTCCCTGGATACCTCGGAAACCTCCAAGACATCCAGGCTTAGCTTCAAGGAGAATGTGG   120
A                   TGTCCCTGGATACCTCGGAAACCTCCAAGACATCCAGGCTTAGCTTCAAGGAGAATGTGG   120
                    ************************************************************

NM_001009808.1      TGATGGTGACAGCCAATGGCAAGATTCTGAAGAAGAGACGGTTGAGTTTAAATCAGTTCA   180
H                   TGATGGTGACAGCCAATGGCAAGATTCTGAAGAAGAGACGGTTGAGTTTAAATCAGTTCA   180
A                   TGATGGTGACAGCCAATGGCAAGATTCTGAAGAAGAGACGGTTGAGTTTAAATCAGTTCA   180
                    ************************************************************

NM_001009808.1      TCACCGATGATGACCTGGAAGCCATTGCCAATGATACCGAAGAAGAAATCATCAAGCCCA   240
H                   TCACCGATGACGACCTGGAAGCCATTGCCAATGATACCGAAGAAGAAATCATCAAGCCCA   240
A                   TCACTGATGATGACCTGGAAGCCATTGCCAATGATACCGAAGAAGAAATCATCAAGCCCA   240
                    **** ***** *************************************************

NM_001009808.1      GATCAGCACATTACAGCTTCCAGAGTAACGTGAAATACAACTTTATGAGAGTCATCCACC   300
H                   GATCAGCACATTACAGCTTCCAGAGTAACGTGAAATACAACTTTATGAGAGTCATCCACC   300
A                   GATCAGCACATTACAGCTTCCAGAGTAACGTGAAATACAACTTTATGAGAGTCATCCACC   300
                    ************************************************************

NM_001009808.1      AGGAATGCATCCTGAACGACGCCCTCAATCAAAGTATAATTCGAGATATGTCAGGTCCAT   360
H                   AGGAATGCATCCTGAACGACGCCCTCAATCAAAGTATAATTCGAGATATGTCAGGTCCAT   360
A                   AGGAATGCATCCTGAACGACGCCCTCAATCAAAGTATAATTCGAGATATGTCAGGTCCAT   360
                    ************************************************************

NM_001009808.1      ACATGACGGCTGCTACATTAAATAATCTGGAGGAGGCAGTGAAATTTGACATGGTTGCTT   420
H                   ACCTGACGGCTGCTACATTAAATAATCTGGAGGAGGCAGTGAAATTTGACATGGTTGCTT   420
A                   ACATGACGGCTGCTACATTAAATAATCTGGAGGAGGCAGTGAAATTTGACATGGTTGCTT   420
                    ** *********************************************************

NM_001009808.1      ATGTATCAGAAGAGGATTCTCAGCTTCCTGTGACTCTAAGAATCTC        466
H                   ATGTATCAGAAGAGGATTCTCAGCTTCCTGTGACTCTAAGAATCTC        466
A                   ATGTATCAGAAGAGGATTCTCAGCTTCCTGTGACTCTAAGAATCTC        466
                    **********************************************
```

Figure S1: Representative Sequence alignment of *IL-1α* gene (466-bp) between healthy (H), and arthritis (A) rams.

```
NM_001009465.2      CAGCCATGGCAACCGTACCTGAACCCATCAATGAAGTGATGGCTTGCTACAGTGATGAGA    60
H                   CAGCCATGGCAACCGTACCTGAACCCATCAATGAAGTGATGGCTTGCTACAGTGATGCGA    60
A                   CAGCCATGGCAACCGTACCTGAACCCATTAATGAAGTGATGGCTTACTACAGTGATGAGA    60
                    **************************** **************** *********** **

NM_001009465.2      ATGAGCTGTTATTTGAGGTTGATGGCCCTAAACAGATGAAGAGCTGCACCCAACACCTGG    120
H                   ATGAGCTGTTATTTGAGGTTGATGGCCCCAAACAGATGAAGAGCTGCACCCAACACCTGG    120
A                   ATGAGCTGTTATTTGAGGTTGATGGCCCTAAACAGATGAAGAGCTGCACCCAACACCTGG    120
                    **************************** *******************************

NM_001009465.2      ACCTCGGCTCCATGGGAGATGGAAACATCCAGCTGCAGATTTCTCACAAGCTCTACAACA    180
H                   ACCTCGGCTCCATGGGAGATGGAAACATCCAGCTGCAGATTTCTCACAAGCTCTACAACA    180
A                   ACCTCGGCTCCATGGGAGATGGAAACATCCAGCTGCAGATTTCTCACAAGCTCTACAACA    180
                    ************************************************************

NM_001009465.2      AAAGCTTCAGGCAGGCAGTGTCGGTCATCGTGGCCATGGAGAAGCTGAGGAGCCGTGCCT    240
H                   AAAGCTTCAGGCAGGCAGTGTCGGTCATCGTGGCCATGGAGAAGCTGAGGAGCCGTGCCT    240
A                   AAAGCTTCAGGCAGGCAGTGTCGGTCATCGTGGCCATGGAGAAGCTGAGGAGCCGTGCCT    240
                    ************************************************************

NM_001009465.2      ACGAACATGTCTTCCGTGATGATGACCTGAGGAGCATCCTTTCATTCATCTTCGAAGAAG    300
H                   ACGAACATGTCTTCCGTGATGATGACCTGAGGAGCATCCTTTCATTCATCTTCGAAGAAG    300
A                   ACGAACATGTCTTCCGTGATGATGACCTGAGGAGCATCCTTTCATTCATCTTCGAAGAAG    300
                    ************************************************************

NM_001009465.2      AGCCTGTCATCTTCGAAACATCCTCCGATGAGCTTCTGTGTGATGCAGCCGTGCAGTCAG    360
H                   AGCCTGTCATCTTCGAAACATCCTCCGATGAGCTTCTGTGTGATGCAGCCGTGCAGTCAG    360
A                   AGCCTGTCATCTTCGAAACATCCTCCGATGAGCTTCTGTGTGATGCAGCCGTGCAGTCAG    360
                    ************************************************************

NM_001009465.2      T       361
H                   T       361
A                   T       361
                    *
```

Figure S2: Representative Sequence alignment of *IL-1β* gene (361-bp) between healthy (H), and arthritis (A) rams.

```
NM_001009392.1      GTAGTTCCTGGGCATTCCCTCCTCTGGTCAGAAACCTGTCCACTGGGCACATAACTTATG    60
H                   GTAGTTCCTGGGCATTCCCTCCTCTGGTCAGAAACCTGTCCACTGGGCACATAACTTATG    60
A                   GTAGTTCCTGGGCATTCCCTCCTCTGGTCAGAAACCTGTCCACTGGGCACATAACTTATG    60
                    ************************************************************

NM_001009392.1      TTGTTCTCTATGAAGAACTAAAAGTATGAGCGTTAGGACACTATTTTATCTTTAATTTAT    120
H                   TTGTTCTCTATGAAGAACTAAAAGTATGAGCGTTAGGACACTATTTTATCTTTAATTTAT    120
A                   TTGTTCTCTATGAAGAACTAAAAGTATGAGCGTTAGGACACTATTTTATCTTTAATTTAT    120
                    ************************************************************

NM_001009392.1      TGATATTTAAATATGTGGTTTTGAGTTAATTTATATACATGATAGGTATTTATATTTTTA    180
H                   TGATATTTAAATATGTGGTTTTGAGTTAATTTATATACATGATAGGTATTTATATTTTTA    180
A                   TGATATTTAAATATGTGGTTTTGAGTTAATTTATATACATGATAGGTATTTATATTTTTA    180
                    ************************************************************

NM_001009392.1      TGAAGTGCCACTTGAAATATTTTATGTATTTGGTTTGAAAAAGCAACGTAAAAATGGCTA    240
H                   TGAAGTGCCACTTGAAATATTTTATGTATTTGGTTTGAAAAAGCAACGTAAAAATGGCTA    240
A                   TGAAGTGCCACTTGAAATATTTTATGTATTTGGTTTGAAAAAGCAACGTAAAAATGGCTA    240
                    ************************************************************

NM_001009392.1      TGCGGCTTGAACGTCCTTATTGTTTTGGAGCCAAATCATTTCTTGAAATGTGTAGGCTTA    300
H                   TGCGGCTTGAACGTCCTTATTGTTTTGGAGCCAAATCATTTCTTGAAATGTGTAGGCTTA    300
A                   TGCGGCTTGAACGTCCTTATTGTTTTGGAGCCAAATCATTTCTTGAAATGTGTAGGCTTA    300
                    ************************************************************

NM_001009392.1      CCTCAAAAAATTTGCTAACTTATGCATATTTTTAAAGGCACATTTATATTGTATTTATAT    360
H                   CCTCAAAAAATTTGCTAACTTATGCATATTTTTAAAGGCATATTTATATTGTATTTATAT    360
A                   CCTCAAAAAATTTGCTAACTTATGCATATTTTTAAAGGCACATTTATATTGTATTTATAT    360
                    **************************************** *******************

NM_001009392.1      GTTTAGGCTG       370
H                   GTTTAGGCTG       370
A                   GTTTAGGCTG       370
                    **********
```

Figure S3: Representative Sequence alignment of *IL-6* gene (370-bp) between healthy (H), and arthritis (A) rams.

```
NM_001024860.1      CCTGCCGGAATACCTGGACTATGCCGAGTCTGGGCAGGTCTACTTTGGGATCATCGCCCT    60
H                   CCTGCCGGAATACCTGGACTATGCCGAGTCTGGGCAGGTCTACTTTGGGATCATCGCCCT    60
A                   CCTGCCGGAATACCTGGACTATGCCGAGTCTGGGCAGGTCTACTTTGGGATCATCGCCCT    60
                    ************************************************************

NM_001024860.1      GTGAGGGCGCAGGACATGCATCCTCTCCCACCTCAGTTACCTTATTATTTACTCCTTCAG    120
H                   GTGAGGGCGCAGGACATGCATCCTCTCCCACCTCAGTTACCTTATTATTTACTCCTTCAG    120
A                   GTGAGGGCGCAGGACATGCATCCTCTCCCACCTCAGTTACCTTATTATTTACTCCTTCAG    120
                    ************************************************************

NM_001024860.1      ACCCTCCTCATCCCCTTCTGGTTTAGAAAGGGAATTAGGGGCTCAGGGCTGGGCTCCAAG    180
H                   ACCCTCCTCATCCCCTTCTGGTTTAGAAAGGGAATTAGGGGCTCAGGGCTGGGCTCCAAG    180
A                   ACCCTCCTCATCCCCTTCTGGTTTAGAAAGGGAATTAGGGGCTCAGGGCTGGGCTCCAAG    180
                    ************************************************************

NM_001024860.1      CGTCCAACTTTAAACAGCTGCACTTAGAAATTAGGGATGTAGGGAAGTGAGGCCTGGACA    240
H                   CGTCCAACTTTAAACAGCTGCACTTAGAAATTAGGGATGTAGGGAAGTGAGGCCTGGACA    240
A                   CGTCCAACTTTAAACAGCTGCACTTAGAAATTAGGGATGTAGGGAAGTGAGGCCTGGACA    240
                    ************************************************************

NM_001024860.1      ACGGGCCACCAACCATCACCAAGGACTGGAACTGGAACTTCCAGAACTCCCTCGGGTCCA    300
H                   ACGGGCCACCAACCATCACCAAGGACTGGAACTGGAACTTCCAGAACTCCCTCGGGTCCA    300
A                   ATGGGCCACCAACCATCACCAAGGACTGGAACTGGAACTTCCAGAACTCCCTCGGGTCCA    300
                    * **********************************************************

NM_001024860.1      CAAGTTTGGGTTCCCGGATGCAACCTGGGACACCCAGAATGCAAGGGCCAGGGTTCTTAC    360
H                   CAAGTTTGGGTTCCCGGATGCAACCTGGGACACCCAGAATGCAAGGGCCAGGGTTCTTAC    360
A                   CAAGTTTGGGTTCCCGGATGCAACCTGGGACACCCAGAATGCAAGGGCCAGGGTTCTTAC    360
                    ************************************************************

NM_001024860.1      CGGAATACTTCGCAACGTTCCTTGAG 386
H                   CGGAATACTTCGCAACGTTCCTTGAG 386
A                   CGGAATACTTCGCAACGTTCCTTGAG 386
                    **************************
```

Figure S4: Representative Sequence alignment of *TNFα* gene (386-bp) between healthy (H), and arthritis (A) rams.

```
NM_001009327.1      CTGAAGACCCTCCGGCTGCGGCTGCGGCGCTGTCATCGTTTTCTGCCCTGCGAAAACAAG   60
H                   CTGAAGACCCTCCGGCTGCGGCTGCGGCGCTGTCATCGTTTTCTGCCCTGCGAAAACAAG   60
A                   CTGAAGACCCTCCGGCTGCGGCTGCGGCGCTGTCATCGCTTTCTGCCCTGCGAAAACAAG   60
                    ************************************** *********************

NM_001009327.1      AGCAAGGCGGTGGAGCAGGTGAAGAGAGTCTTCAATATGCTCCAAGAGAGGGGTGTCTAC   120
H                   AGCAAGGCGGTGGAGCAGGTGAAGAGAGTCTTCAATATGCTCCAAGAGAGGGGTGTCTAC   120
A                   AGCAAGGCGGTGGAGCAGGTGAAGAGAGTCTTCAATATGCTCCAAGAGAGGGGTGTCTAC   120
                    ************************************************************

NM_001009327.1      AAAGCCATGAGTGAGTTTGACATCTTCATCAACTACATAGAATCCTACATGACAACGAAG   180
H                   AAAGCCATGAGTGAGTTTGACATCTTCATCAACTACATAGAATCCTACATGACAACGAAG   180
A                   AAAGCCATGAGTGAGTTTGACATCTTCATCAACTACATAGAATCCTACATGACAACGAAG   180
                    ************************************************************

NM_001009327.1      ATGTAAAACTGAAGCATTCTAGGGAAGGAGACCTCCAGGATGGTGACTCGACTAGACTCC   240
H                   ATGTAAAACTGAAGCATTCTAGGGAAGGAGACCTCCAGGATGGTGACTCGACTAGACTCC   240
A                   ATGTAAAACTGAAGCATTCTAGGGAAGGAGACCTCCAGCATGGTGACTCGACTAGACTCC   240
                    ************************************** *********************

NM_001009327.1      CCGACATAAACCTCTGAAATCCGACCCAGGGTTCTGGGAGAGCAGAGCCAGCTCCCTGGA   300
H                   CCGACATAAACCTCTGAAATCCGACCCAGGGTTCTGGGAGAGCAGAGCCAGCTCCCTGGA   300
A                   CCGACATAAACCTCTGAAATCCGACCCAGGGTTCTGGGAGAGCAGAGCCAGCTCCCTGGA   300
                    ************************************************************

NM_001009327.1      GACCTCTACTGTGCCTCTCCCCTAGAGTATTTATTACCTCTGATACCTCAGCTCCCACAT   360
H                   GACCTCTACTGTGCCTCTCCCCTAGAGTATTTATTACCTCTGATACCTCAGCTCCCACAT   360
A                   GACCTCTACTGTGCCTCTCCCCTAGAGTATTTATTACCTCTGATACCTCAGCTCCCACAT   360
                    ************************************************************

NM_001009327.1      CTATTTATTTACTGAGCTTCTCTGTGA         387
H                   CTATTTATTTACTGAGCTTCTCTGTGA         387
A                   CTATTTATTTACTGAGCTTCTCTGTGA         387
                    ***************************
```

Figure S5: Representative Sequence alignment of *IL-10* gene (387-bp) between healthy (H), and arthritis (A) rams.

```
XM_060416845.1      ATCCACCTGCACGCACACAGCCTGGTGGGAAAACATTGTGAGGATGGCGTCTGCACTGTG   60
H                   ATCCACCTGCACGCACACAGCCTGGTGGGAAAACATTGTGAGGATGGCGTCTGCACTGTG   60
A                   ATCCACCTGCACGCACACAGCCTGGTGGGAAAACATTGTGAGGATGGCGTCTGCACTGTG   60
                    ************************************************************

XM_060416845.1      ACAGCCGGGCCCAAGGACATGGTGGTCGGCTTTGCAAACCTGGGTATACTTCATGTGACA   120
H                   ACAGCTGGGCCCAAGGACATGGTGGTCGGCTTTGCAAACCTGGGTATACTTCATGTGACA   120
A                   ACAGCCGGGCCCAAGGACATGGTGGTCGGCTTTGCAAACCTGGGTATACTTCATGTGACA   120
                    ***** ******************************************************

XM_060416845.1      AAGAAAAAAGTATTTGAAACACTGGAAGCACGAATGACAGACGCCTGTGTAAGGGGCTAT   180
H                   AAGAAAAAAGTATTTGAAACACTGGAAGCACGAATGACAGACGCCTGTGTAAGGGGCTAT   180
A                   AAGAAAAAAGTATTTGAAACACTGGAAGCACGAATGACAGACGCCTGTGTAAGGGGCTAT   180
                    ************************************************************

XM_060416845.1      AATCCCGGGCTTTTGGTGCATCCTGATCTTGCCTATTTGCAGGCAGAAGGTGGAGGAGAC   240
H                   AATCCCGGGCTTTTGGTGCATCCTGATCTTGCCTATTTGCAGGCAGAAGGTGGAGGAGAC   240
A                   AATCCCGGGCTTTTGGTGCATCCTGATCTTGCCTATTTGCAGGCAGAAGGTGGAGGAGAC   240
                    ************************************************************

XM_060416845.1      CGGCAGCTCACAGATCGGGAAAAAGAAATCATCCGCCAGGCAGCTCTTCAGCAGACGAAG   300
H                   CGGCAGCTCACAGATCGGGAAAAGGAAATCATCCGCCAGGCAGCTCTTCAGCAGACGAAG   300
A                   CGGCAGCTCACAGATCGGGAAAAAGAAATCATCCGCCAGGCAGCTCTTCAGCAGACGAAG   300
                    ************************ ***********************************

XM_060416845.1      GAGATGGACCTCAGCGTGGTACGGCTCATGTTTACAGCTTTCCTTCCAGACAGCACCGGC   360
H                   GAGATGGACCTCAGCGTGGTACGGCTCATGTTTACAGCTTTCCTTCCAGACAGCACCGGC   360
A                   GAGATGGACCTCAGCGTGGTACGGCTCATGTTTACAGCTTTCCTTCCAGACAGCACCGGC   360
                    ************************************************************

XM_060416845.1      AGCTTCACGAGGCGTCTGGAACCCGTGGTGTCGGACGCCATCTATGACAGC   411
H                   AGCTTCACGAGGCGTCTGGAACCCGTGGTGTCGGACGCCATCTATGACAGC   411
A                   AGCTTCACGAGGCGTCTGGAACCCGTGGTGTCGGACGCCATCTATGACAGC   411
                    ***************************************************
```

Figure S6: Representative Sequence alignment of *NFKB* gene (411-bp) between healthy (H), and arthritis (A) rams.

```
XM_042247241.2      AGAGGCAGCTCCTGGGGACTCCTGGGACCACAGCCGAGACGAGGCTAGCTGGAGGGAAGT    60
H                   AGAGGCAGCTCCTGGGGACTCCTGGGACCACAGCCGAAACGAGGCTAGCTGGAGGGAAGT    60
A                   AGAGGCAGCTCCTGGGGACTCCTGGGACCACAGCCTAGACGAGGCTAGCTGGAGGGAAGT    60
                    *********************************** * **********************

XM_042247241.2      GAGAGGTGACCTCGGCCTGGGCCCGGTCGTGAGACCCTCCGCCACCCCTGCCCTGGACGG    120
H                   GAGAGGTGACCTCGGCCTGGGCCCGGTCGTGAGACCCTCCGCCACCCCTGCCCTGGACGG    120
A                   GAGAGGTGACCTCGGCCTGGGCCCGGTCGTGAGACCCTCCGCCACCCCTGCCCTGGACGG    120
                    ************************************************************

XM_042247241.2      ACAGCCATGGCCAGGGCGCAGCAGCTTCGGGCTGAGAGCGACTTCGACCAGCTTCCTGAC    180
H                   ACAGCCATGGCCAGGGCGCAGCAGCTTCGGGCTGAGAGCGACTTCGACCAGCTTCCTGAC    180
A                   ACAGCCATGGCCAGGGCGCAGCAGCTTCGGGCTGAGAGCGACTTCGACCAGCTTCCTGAC    180
                    ************************************************************

XM_042247241.2      GACATTGCCATCTCAGCCAACATCGCTGACATTGAGGAGAAGAGAGGCTTCACCAGCCAC    240
H                   GACATTGCCATCTCAGCCAACATCGCTGACATTGAGGAGAAGAGAGGCTTCACCAGCCAC    240
A                   GACATTGCCATCTCAGCCAACATCGCTGACATTGAGGAGAAGAGAGGCTTCACCAGCCAC    240
                    ************************************************************

XM_042247241.2      TTCGTTTTTGTCATCGAGGTGAAGACAAAAGGGGGGTCCAAGTACCTCATCTACCGCCGC    300
H                   TTCGTTTTTGTCATCGAGGTGAAGACAAAAGGGGGGTCCAAGTACCTCATCTACCGCCGC    300
A                   TTTGTTTTTGTCATCGAGGTAAAGACGAAAGGGGGGTCCAAGTACCTCATCTACCGCCGC    300
                    ** ***************** ***** *********************************

XM_042247241.2      TACCGCCAGTTCTATGCCTTGCAGAGCAAGCTGGAGGAGCGCTTC          345
H                   TACCGCCAGTTCTATGCCTTGCAGAGCAAGCTGGAGGAGCGCTTC          345
A                   TACCGCCAGTTCTATGCCTTGCAGAGCAAGCTGGAGGAGCGCTTC          345
                    *********************************************
```

Figure S7: Representative Sequence alignment of *NCF4* gene (345-bp) between healthy (H), and arthritis (A) rams.

```
XM_060415483.1      AGCACTGGCTGGCTTGCAGGTGTGGGTCCCTCCAGGAGGAATGGCTCAGTGGCTGCACCT   60
H                   AGCACTGGCTGGCTTGCAGGTGTGGGTCCCTCCAGGAGGAATGGCTCAGTGGCTGCACCT   60
A                   AGCACTGGCTGGCTTGCAGGTGTGGGTCCCTCCAGGAGGAATGGCTCAGTGGCTGCACCT   60
                    ************************************************************

XM_060415483.1      GTTTTCCTAGAAATGGTGCATGGGGGGCACTGCAAGGTTCGAAGGCAACCCTGGGGTTTT   120
H                   GTTTTCCTAGAAATGGTGCATGGGGGGCACTGCAAGGTTCGAAGGCAACCCTGGGGTTTT   120
A                   GTTTTCCTAGAAATGGTGCATGGGGGGCACTGCAAGGTTCGAAGGCAACCCTGGGGTTTT   120
                    ************************************************************

XM_060415483.1      CTGGGCCTGCCTCTGTCCCCTACTGTGACGTGCCTTAGCCAGTCTTCCAGTCGGCACCAG   180
H                   CTGGCCCTGCCTCTGTCCCCTACTGTAACGTGCCTTAGCCAGTCTTCCAGTCGGCACCAG   180
A                   CTGGGCCTGCCTCTGTCCCCTACTGTGACGTGCCTTAGCCAGTCTTCCAGTCGGCACCAG   180
                    **** ********************* *********************************

XM_060415483.1      CCCTGTGCAGTGCTGGACCGGGCCCAGGAAGCAAACTCCCCGTTGTCTTCCCGCTGGCCC   240
H                   CCCTGTGCAGTGCTGGACCGGGCCCAGGAAGCAAACTCCCCGTTGTCTTCCCGCTGGCCC   240
A                   CCCTGTGCAGTGCTGGACCGGGCCCAGGAAGCAAACTCCCCGTTGTCTTCCCGCTGGCCC   240
                    ************************************************************

XM_060415483.1      TCCAAGTTAGGTCCCAATAGGAGGGGGCTGACTGCTGCAGGATCCTCTGCCTGCCTGCCT   300
H                   TCCAAGTTAGGTCCCAATAGGAGGGGGCTGACTGCTGCAGGATCCTCTGCCTGCCTGCCT   300
A                   TCCAAGTTAGGTCCCAATAGGAGGGGGCTGACTGCTGCAGGATCCTCTGCCTGCCTGCCT   300
                    ************************************************************

XM_060415483.1      TCTCCTTGTGGCTCCACCCAAGGCCTGTGCTGCTACTGGGGGTGGCCTGCTGTGCCAGGG   360
H                   TCTCCTTGTGGCTCCACCCAAGGCCTGTGCTGCTACCGGGGGTGGCCTGCTGTGCCAGGG   360
A                   TCTCCTTGTGGCTCCACCCAAGGCCTGTGCTGCTACTGGGGGTGGCCTGCTGTGCCAGGG   360
                    ************************************ ***********************

XM_060415483.1      GCCAGGGGGAACAAGACGTGGCTGCAGTGTTCTTGCCAGAACAGTCAC     408
H                   GCCAGGGGGAACAAGACGTGGCTGCAGTGTTCTTGCCAGAACAGTCAC     408
A                   GCCAGGGGGAACAAGACGTGGCTGCAGTGTTCTTGCCAGAACAGTCAC     408
                    ************************************************
```

Figure S8: Representative Sequence alignment of *TMED1* gene (408-bp) between healthy (H), and arthritis (A) rams.

```
XM_042257020.1      GAGGCTTGTTCGTGGTGAGGCTGTCCCAGCTGACCCCGGAGGATGAGGGGCGCTACCGCT    60
H                   GAGGCTTGTTCGTGGTGAGGCTGTCCCAGCTGACCCCGGAGGATGAGGGGCGCTACCGCT    60
A                   GAGGCTTGTTCGTGGTGAGGCTGTCCCAGCTGACCCCGGAGGATGAGGGGCGCTACCGCT    60
                    ************************************************************

XM_042257020.1      GCGGCCTCGGGAGCTCAAACAACGCGCTTTTCTTCAGCATGAACCTGACGGTCTCTCCAG    120
H                   GCGGCCTCGGGAGCTCAAACAACGCGCTTTTCTTCAGCATGAACCTGACGGTCTCTCCAG    120
A                   GCGGCCTCGGGAGCTCAAACAACGCGCTTTTCTTCAGCATGAACCTGACGGTCTCTCCAG    120
                    ************************************************************

XM_042257020.1      GTCTTTCCAGAACCATCCCCACAGCCACTCTGGCCTATGGTGAGCTCATCACAGGATCCT    180
H                   GTCTTTCCAGAACCATCCCCACAGCCACTCTGGCCTATGGTGAGCTCATCACAGGATCCT    180
A                   GTCTTTCCAGAACCATCCCCACAGCCACTCTGGCCTATGGTGAGCTCATCACAGGATCCT    180
                    ************************************************************

XM_042257020.1      TTGAAATAGCATCACCCCCAGCAGCCAAAAGATGCACACGAGGAAACACCCAGACGACAG    240
H                   TTGAAATAGCATCACCCCCAGCAGCCAAAAGATGCACACGAGGAAACACCCAGACGACAG    240
A                   TTGAAATAGCATCACCCCCAGCAGCCAAAAGATGCACACGAGGAAACACCCAGACGACAG    240
                    ************************************************************

XM_042257020.1      GAAGACAGAGGACAGGATGGGATACGGCTGCCCTGACTCCAGGATCCAGGAAAACCATGG    300
H                   GAAGACAGAGGACAGGATGGGATACGGCTGCCCTGACTCCAGGATCCAGGAAAACCATGG    300
A                   GAAGACAGAGGACAGGATGGGATACGGTTGCCCTGACTCCAGGATCCAGGAAAACCATGG    300
                    *************************** ********************************

XM_042257020.1      CTTCAGCCACGGGAGAGCAAACCCCAGGAGCAGCTGCGGTACTGGCTCCAGGGACAGGCA    360
H                   CTTCAGCCACGGGAGAGCAAACCCCAGGAGCAGCTGCGGTACTGGCTCCAGGGACAGGCA    360
A                   CTTCAGCCACGGGAGAGCAAACCCCAGGAGCAGCTGCGGTACTGGCTCCAGGGACAGGCA    360
                    ************************************************************

XM_042257020.1      GCCAGGCAGAGGGCTCCG          378
H                   GCCAGGCAGAGGGCTCCG          378
A                   GCCAGGCAGAGGGCTCCG          378
                    ******************
```

Figure S9: Representative Sequence alignment of *FCAMR* gene (378-bp) between healthy (H), and arthritis (A) rams.

```
AF223942.1      CAGGAACCTACCAGCTGACGGGAGATGAGCTCATCTTCGCCACCAAGCAGGCCTGGCGCA      60
H               CAGGAACCTACCAGCTGACGGGAGATGAGCTCATCTTTGCCACCAAGCAGGCCTGGCGCA      60
A               CAGGAACCTACCAGCTGACGGGAGATGAGCTCATCTTCGCCACCAAGCAGGCCTGGCGCA      60
                ************************************* **********************

AF223942.1      ACGCCCCCCGCTGCATCGGGAGGATCCAGTGGTCGAACCTGCAGGTCTTTGACGCCCGGA      120
H               ACGCCCCCCGCTGCATTGGGAGGATCCAGTGGTCGAACCTGCAGGTCTTTGACGCCCGGA      120
A               ACGCCCCCCGCTGCATCGGGAGGATCCAGTGGTCGAACCTGCAGGTCTTTGACGCCCGGA      120
                **************** *******************************************

AF223942.1      GCTGTTCCACGGCCCAGGAAATGTTCGAACACATCTGTAGACACGTGCGTTACGCCACCA      180
H               GCTGTTCCACGGCCCAGGAAATGTTCGAACACATCTGTAGACACGTGCGTTACGCCACCA      180
A               GCTGTTCCACGGCCCAGGAAATGTTCGAACACATCTGTAGACACGTGCGTTACGCCACCA      180
                ************************************************************

AF223942.1      ACAACGGCAACATCAGGTCGGCCATCACCGTGTTCCCCCAGCGGAGCGATGGGAAGCATG      240
H               ACAACGGCAACATCAGGTCGGCCATCACCGTGTTCCCCCAGCGGAGCGATGGGAAGCATG      240
A               ACAACGGCAACATCAGGTCGGCCATCACCGTGTTCCCCCAGCGGAGCGATGGGAAGCATG      240
                ************************************************************

AF223942.1      ACTTCCGGGTCTGGAATGCCCAGCTCATCCGCTATGCCGGCTACCAGATGCCAGATGGCA      300
H               ACTTCCGGGTCTGGAATGCCCAGCTCATCCGCTATGCCGGCTACCAGATGCCAGATGGCA      300
A               ACTTCCGGGTCTGGAATGCCCAGCTCATCCGCTATGCCGGCTACCAGATGCCAGATGGCA      300
                ************************************************************

AF223942.1      GCATCAGAGGGGACCCCGCCAGTGTGGAGTTCACACAGCTGTGCATCGACCTGGGCTGGA      360
H               GCATCAGAGGGGACCCCGCCAGTGTGGAGTTCACACAGCTGTGCATCGACCTGGGCTGGA      360
A               GCATCAGAGGGGACCCCGCCAGTGTGGAGTTCACACAGCTGTGCATCGACCTGGGCTGGA      360
                ************************************************************

AF223942.1      AGCCCAAGTTCAGCCGCTTCGACGTGTTGCCTCTAGTCCTGCAGGCTGATGGCCGCGACC      420
H               AGCCCAAGTTCAGCCGCTTCGACGTGTTGCCTCTAGTCCTGCAGGCTGATGGCCGCGACC      420
A               AGCCCAAGTTCAGCCGCTTCGACGTGTTGCCTCTAGTCCTGCAGGCTGACGGCCGCGACC      420
                ************************************************* **********

AF223942.1      AGCCCAAGTTCAGCCGCTTCGACGTGTTGCCTCTAGTCCTGCAGGCTGACGGCCGCGACC      480
H               AGCCCAAGTTCAGCCGCTTCGACGTGTTGCCTCTAGTCCTGCAGGCTGACGGCCGCGACC      480
A               AGCCCAAGTTCAGCCGCTTCGACGTGTTGCCTCTAGTCCTGCAGGCTGACGGCCGCGACC      480
                ************************************************************
```

Figure S10: Representative Sequence alignment of *iNOS* gene (480-bp) between healthy (H), and arthritis (A) rams.

```
XM_027970902.2      ATCCGCGACATGCACGCCAAGGTGACAGAGATCTGGCAGGAGATGATGCAGCGGCAGGCG   60
H                   ATCCGCGACATGCACGCCAAGGTGACAGAGATCTGGCAGGAGATGATGCAGCGGCAGGCG   60
A                   ATCCGCGACATGCACGCCAAGGTGACAGAGATCTGGCAGGAGATGATGCAGCGGCAGGCG   60
                    ************************************************************

XM_027970902.2      GCGGCCATCGACCCGGACGCGGCGCTCCATGCGGTCTGCCGGGTGCTGCCGTCGGCCACG   120
H                   GCGGCCATCGACCCGGACGCGGCGCTCCATGCGGTCTGCCGGGTGCTGCCGTCGGCCACG   120
A                   GCGGCCATCGACCCGGACGCGGCGCTCCATGCGGTCTGCCGGGTGCTGCCGTCGGCCACG   120
                    ************************************************************

XM_027970902.2      CTGGAGGCGGAGCAGCCCCGGGTCAGCGGCCTCGTGCTCTTCCGGCAGCTCCGGCCTGGC   180
H                   CTGGAGGCGGAGCAGCCCCGGGTCAGCGGCCTCGTGCTCTTCCGGCAGCTCCGGCCTGGC   180
A                   CTGGAGGCGGAGCAGCCCCGGGTCAGGGGCCTCGTGCTCTTCCGGCAGCTCCGGCCTGGC   180
                    ************************** *********************************

XM_027970902.2      GCCCTGCTGGAGGCCTTCTTCCACCTGGAGGGCTTCCCGAACGAGCCCAACGGCACAAGC   240
H                   GCCCTGCTGGAGGCCTTCTTCCACCTGGAGGGCTTCCCGAACGAGCCCAACGGCACAAGC   240
A                   GCCCTGCTGGAGGCCTTCTTCCACCTGGAGGGCTTCCCGAACGAGCCCAACGGCACAAGC   240
                    ************************************************************

XM_027970902.2      CGCGCCATCCACGTGCACCAGTTTGGGGACCTGAGCCAGGGCTGCGACTCCACCGGGCCG   300
H                   CGCGCCATCCACGTGCACCAGTTTGGGGACCTGAGCCAGGGCTGCGACTCCACCGGGCCG   300
A                   CGCGCCATCCACGTGCACCAGTTTGGGGACCTGAGCCAGGGCTGCGACTCCACCGGGCCG   300
                    ************************************************************

XM_027970902.2      CACTACAACCCGATGTCCGTGCTGCACCCGCAGCACCCGGGCGACTTTGGCAACTTCGCC   360
H                   CACTACAACCCGATGTCCGTGCTGCACCCGCAGCACCCGGGCGACTTTGGCAACTTCGCC   360
A                   CACTACAACCCGATGTCCGTGCTGCACCCGCAGCACCCGGGCGACTTTGGCAACTTCGCC   360
                    ************************************************************

XM_027970902.2      GTGCGAGATGGCCAGGTCTGG     381
H                   GTGCGAGATGGCCAGGTCTGG     381
A                   GTGCGAGATGGCCAGGTCTGG     381
                    *********************
```

Figure S11: Representative Sequence alignment of *SOD* gene (381-bp) between healthy (H), and arthritis (A) rams.

```
XM_060400055.1      CTGATGTCCTGACCACTGGCGCCGGTAATCCAGTAGGAGACAAACTCAATGTTCTGACGG   60
H                   CTGATGTCCTGACCACTGGCGCCGGTAATCCAGTAGGAGACAAACTCAATGTTCTGACGG   60
A                   CTGATGTCCTGACCACTGGCGCCGGTAATCCAGTAGGAGACAAACTCAATGTTCTGACGG   60
                    ************************************************************

XM_060400055.1      TAGGGCCCCGAGGGCCCCTTCTCGTCCAGGATGTGGTTTTCACTGATGAAATGGCTCACT   120
H                   TAGGGCCCCGAGGGCCCCTTCTCGTCCAGGATGTGGTTTTCACTGATGAAATGGCTCACT   120
A                   TAGGGCCCCGAGGGCCCCTTCTCGTCCAGGATGTGGTTTTCACTGATGAAATGGCTCACT   120
                    ************************************************************

XM_060400055.1      TTGACCGGGAGAGAATTCCTGAGAGAGTCGTGCACGCCAAAGGAGCAGGGGCTTTTGGCT   180
H                   TTGACCGGGAGAGAATTCCTGAGAGAGTCGTGCACGCCAAAGGAGCAGGGGCTTTTGGCT   180
A                   TTGACCGGGAGAGAATTCCTGAGAGAGTCGTGCATGCCAAAGGAGCAGGGGCTTTTGGCT   180
                    ********************************** *************************

XM_060400055.1      ACTTTGAGGTCACACATGACATTACCAGATACTCCAAGGCGAAGGTGTTTGAGCATATTG   240
H                   ACTTTGAGGTCACACATGACATTACCAGATACTCCAAGGCGAAGGTGTTTGAGCATATTG   240
A                   ACTTTGAGGTCACACATGACATTACCAGATACTCCAAGGCGAAGGTGTTTGAGCATATTG   240
                    ************************************************************

XM_060400055.1      GAAAGAGGACGCCCATTGCAGTTCGCTTCTCCACTGTTGCTGGAGAATCAGGCTCAGCTG   300
H                   GAAAGAGGACGCCCATTGCAGTTCGCTTCTCCACTGTTGCTGGAGAATCAGGCTCAGCTG   300
A                   GAAAGAGGACGCCCATTGCAGTTCGCTTCTCCACTGTTGCTGGAGAATCAGGCTCAGCTG   300
                    ************************************************************

XM_060400055.1      ACACAGTGCGTGACCCTCGTGGCTTTGCAGTGAAATTTTACACAGAAGATGGTAATTGGG   360
H                   ACACAGTGCGTGACCCTCGTGGCTTTGCAGTGAAATTTTACACAGAAGATGGTAATTGGG   360
A                   ACACAGTGCGTGACCCTCGTGGCTTTGCAGTGAAATTTTACACAGAAGATGGTAATTGGG   360
                    ************************************************************

XM_060400055.1      ATCTTGTTGGAAATAACACCCCCATTTTCTTCATCAGGGATGCTCTACTGTTTCCGTCCT   420
H                   ATCTTGTTGGAAATAACACCCCCATTTTCTTCATCAGGGATGCTCTACTGTTTCCGTCCT   420
A                   ATCTTGTTGGAAATAACACCCCCATTTTCTTCATCAGGGATGCTCTACTGTTTCCGTCCT   420
                    ************************************************************

XM_060400055.1      TTATCCACAGCCAGAAGAGAAACCCTCAGACACACCTGAAGGATCCGGACATG 473
H                   TTATCCACAGCCAGAAGAGAAACCCTCAGACACACCTGAAGGATCCGGACATG 473
A                   TTATCCACAGCCAGAAGAGAAACCCTCAGACACACCTGAAGGATCCGGACATG 473
                    *****************************************************
```

Figure S12: Representative Sequence alignment of *CAT* gene (473-bp) between healthy (H), and arthritis (A) rams.

```
XM_004018462.5      AGCTCACTGCTCTCAACTTGGATCTCATACCATGTGCGCCGCTCAGCGCTCGGCGGCCGC    60
H                   AGCTCACTGCTCTCAACTTGGATCTCATACCATGTGCGCCGCTCAGCGCTCGGCGGCCGC    60
A                   AGCTCACTGCTCTCAACTTGGATCTCATACCATGTGCGCCGCTCAGCGCTCGGCGGCCGC    60
                    ************************************************************

XM_004018462.5      CCTGGCGGCGGCGGCCCCGCGCACGGTGTACGCCTTCTCCGCGCGTCCTCTGGCCGGCGG    120
H                   CCTGGCGGCGGCGGCCCCGCGCACGGTGTACGCCTTCTCCGCGCGTCCTCTGGCCGGCGG    120
A                   CCTGGCGGCGGCGGCCCCGCGCACGGTGTACGCCTTCTCCGCGCGTCCTCTGGCCGGCGG    120
                    ************************************************************

XM_004018462.5      GGAGCCCTTCCACCTGGCCTCCCTGCGGGGCAAGGTGCTGCTCATTGAGAACGTAGCATC    180
H                   GGAGCCCTTCCACCTGGCCTCCCTGCGGGGCAAGGTGCTGCTCATTGAGAACGTAGCATC    180
A                   GGAGCCCTTCCACCTGGCCTCCCTGCGGGGCAAGGTGCTGCTCATTGAGAACGTAGCATC    180
                    ************************************************************

XM_004018462.5      GCTCTGAGGCACCACGGTGCGGGACTACACCCAGATGAATGACCTGCAGCGGCGCCTCGG    240
H                   GCTCTGAGGCACAACGGTGCGGGACTACACCCAGATGAATGACCTGCAGCGGCGCCTCGG    240
A                   GCTCTGAGGCACCACGGTGCGGGACTACACCCAGATGAATGACCTGCAGCGGCGCCTCGG    240
                    ************ ***********************************************

XM_004018462.5      ACCCCGGGGCCTGGTCGTACTCGGCTTCCCGTGCAACCAGTTTGGGCATCAGGAAAACGC    300
H                   ACCCCGGGGCCTGGTCGTACTCGGCTTCCCGTGCAACCAGTTTGGGCATCAGGAAAATGC    300
A                   ACCCCGGGGCCTGGTCGTACTCGGCTTCCCGTGCAACCAGTTTGGGCATCAGGAAAACGC    300
                    ********************************************************* **

XM_004018462.5      CAAGAACGAGGAGATCCTGAATTGCCTGAAGTACGTCCGACCAGGCGGTGGGTTCGAGCC    360
H                   CAAGAACGAGGAGATCCTGAATTGCCTGAAGTACGTCCGACCAGGCGGTGGGTTCGAGCC    360
A                   CAAGAACGAGGAGATCCTGAATTGCCTGAAGTACGTCCGACCAGGCGGTGGGTTCGAGCC    360
                    ************************************************************

XM_004018462.5      CAATTTCATGCTCTTCGAAAAGTGCGAGGTGAATGGCGAGAAGGCGCATCCGCTCT        416
H                   CAATTTCATGCTCTTCGAAAAGTGCGAGGTGAATGGCGAGAAGGCGCATCCGCTCT        416
A                   CAATTTCATGCTCTTCGAAAAGTGCGAGGTGAATGGCGAGAAGGCGCATCCGCTCT        416
                    ********************************************************
```

Figure S13: Representative Sequence alignment of *GPX* gene (416-bp) between healthy (H), and arthritis (A) rams.

```
NM_001009429.1      CGTTGCTGTCGGAGGCGTAGTCACCGCCGCAGCCACCACCTCCTCCTCAATCATGCCGAA    60
H                   CGTTGCTGTCGGAGGCGTAGTCACCGCCGCAGCCACCACCTCCTCCTCAATCATGCCGAA    60
A                   CGTTGCTGTCGGAGGCGTAGTCACCGCCGCAGCCACCACCTCCTCCTCAATCATGCCGAA    60
                    ************************************************************

NM_001009429.1      GCACGAGTTCTCCGTGGATATGACCTGTGAAGGCTGCTCTAACGCAGTCACTCGAGTCCT    120
H                   GCACGAGTTCTCCGTGGATATGACCTGTGAAGGCTGCTCTAACGCAGTCACTCGAGTCCT    120
A                   GCACGAGTTCTCCGTGGATATGACCTGTGAAGGCTGCTCTAACGCAGTCACTCGAGTCCT    120
                    ************************************************************

NM_001009429.1      CAACAAGCTAGGAGGAGTTCAATTTGACATTGACCTGCCCAACAAAAAGGTCTGCATCAA    180
H                   CAACAAGCTAGGAGGAGTTCAATTTGACATTGACCTGCCCAACAAAAAGGTCTGCATCAA    180
A                   CAACAAGCTAGGAGGAGTTCAATTTGACATTGACCTGCCCAACAAAAAGGTCTGCATCAA    180
                    ************************************************************

NM_001009429.1      CTCTGAGCACAGCGTGGACACTTTGCTGGAGACCCTGGGGAAAACAGGAAAGGCTGTCTC    240
H                   CTCTGAGCACAGCGTGGACACTTTGCTGGAGACCCTGGGGAAAACAGGAAAGGCTGTCTC    240
A                   CTCTGAGCACAGCGTGGACACTTTGCTGGAGACCCTGGGGAAAACAGGAAAGGCTGTCTC    240
                    ************************************************************

NM_001009429.1      CTACCTTGGCCCCAAGTAGAGAGGCCCCGTCCAGCAGCCTGCAGGATGGACCAGCATGGG    300
H                   CTACCTTGGCCCCAAGTAGAGAGGCCCCGTCCAGCAGCCTGCAGGATGGACCAGCATGGG    300
A                   CTACCTTGGCCCCAAGTAGAGAGGCCCCGTCCAGCAGCCTGCAGGATGGACCAGCATGGG    300
                    ************************************************************

NM_001009429.1      CAGGACGCTGATCCTCTCCTGCCTTCCAGACAGACCTAGGACCTGGCAATCCCGCTCAGC    360
H                   CAGGACGCTGATCCTCTCCTGCCTTCCAGACAGACCTAGGACCTGGCAATCCCGCTCAGC    360
A                   CAGGACGCTGATCCTCTCCTGCCTTCCAGACAGACCTAGGACCTGGCAATCCCGCTCAGC    360
                    ************************************************************

NM_001009429.1      AATGGTAGTTCCTGCGGAGACCGTCACTTGCCCTGCTCCTCTGTAGCTTCCCTGCAATAA    420
H                   AATGGTAGTTCCTGCGGAGACCGTCACTTGCCCTGCTCCTCTGTAGCTTCCCTGCAATAA    420
A                   AATGGTAGTTCCTGCGGAGACCCTCACTTGCCCTGCTCCTCTGTAGCTTCCCTGCAATAA    420
                    ********************** *************************************

NM_001009429.1      AGTCAAGCCG      430
H                   AGTCAAGCCG      430
A                   AGTCAAGCCG      430
                    **********
```

Figure S14: Representative Sequence alignment of *ATOX1* gene (430-bp) between healthy (H), and arthritis (A) rams.

```
XM_027971062.3      ATGCGCGTAGAGGCCTCGGCGGAGCGGTGCGAGCGTTCCTGGTCCGTGAGGCCGGCGTTT    60
H                   ATGCGCGTAGAGGCCTCGGCGGAGCGGTGCGAGCGTTCCTGGTCCGTGAGGCCGGCGTTT    60
A                   ATGCGCGTAGAGGCCTCGGCGGAGCGGTGCGAGCGTTCCTGGTCCGTGAGGCCGGCGTTT    60
                    ************************************************************

XM_027971062.3      CGGTCCCAGCCCTGGCGCGGAGATGCTGTGCCGGCTCAGCGTTAGGTGGTTGCGGCCCCG    120
H                   CGGTCCCAGCCCTGGCGCGGAGATGCTGTGCCGGCTCAGCGTTAGGTGGCTGCGGCCCCG    120
A                   CGGTCCCAGCCCTGGCGCGGAGATGCTGTGCCGGCTCAGCGTTAGGTGGTTGCGGCCCCG    120
                    ************************************************* **********

XM_027971062.3      GCCTGCCCTGCAGGTCCGTGTCTTGGACCCATCGCTCGCAGGGGCCCCAGCGGGCGGCGC    180
H                   GCCTGCCCTGCAGGTCCGTGTCTTGGACCCATCGCTCGCAGGGGCCCCAGCGGGCGGCGC    180
A                   GCCTGCCCTGCAGGTCCGTGTCTTGGACCCATCGCTCGCAGGGGCCCCAGCGGGCGGCGC    180
                    ************************************************************

XM_027971062.3      CAAGAGCTCCGCTCTGCCAGTGTGGGCGGTGGCATCGGTCTCCGCAGTTGGCCCGAGCGG    240
H                   CAAGAGCTCCGCTCTGCCAGTGTGGGCGGTGGCATCGGTCTCCGCAGTTGGCCCGAGCGG    240
A                   CAAGAGCTCCGCTCTGCCAGTGTGGGCGGTGGCATCGGTCTCCGCAGTTGGCCCGAGCGG    240
                    ************************************************************

XM_027971062.3      CGCGGGAGGCTGGTACGAGGCCTTGGCTACCTCGGCGCCGGTGCAGAGCGCAGAGGACGT    300
H                   CGCGGGAGGCTGGTACGAGGCCTTGGCTACCTCGGCGCCGGTGCAGAGCGCAGAGGACGT    300
A                   CGCGGGAGGCTGGTACGAGGCCTTGGCTACCTCGGCGCCGGTGCAGAGCGCAGAGGACGT    300
                    ************************************************************

XM_027971062.3      ACTGCTCTTCGCCCACACCGCCTCGGGCCTGCCCTGGTGGGGCAGCATTCTCCTCACCAC    360
H                   ACTGCTCTTCGCCCACACCGCCTCGGGCCTGCCCTGGTGGGGCAGCATTCTCCTCACCAC    360
A                   ACTGCTCTTCGCCCACACCGCCTCGGGCCTGCCCTGGTGGGGCAGCATTCTCCTCACCAC    360
                    ************************************************************

XM_027971062.3      CGTGGCCCTGCGCGGGGCCGTCACGCTACCCCTGGCTGCCTACCAGCACTACATC         415
H                   CGTGGCCCTGCGCGGGGCCGTCACGCTACCCCTGGCTGCCTACCAGCACTACATC         415
A                   CGTGGCCCTGCGCGGGGCCGTCACGCTACCCCTGGCTGCCTACCAGCACTACATC         415
                    *******************************************************
```

Figure S15: Representative Sequence alignment of *COX18* gene (415-bp) between healthy (H), and arthritis (A) rams.
